# Risk indicators for dental caries, and gingivitis among 6–11-year-old children in Nigeria: a household-based survey

**DOI:** 10.1186/s12903-022-02470-1

**Published:** 2022-11-03

**Authors:** Morenike Oluwatoyin Folayan, Abiola Adetokunbo Adeniyi, Olaniyi Arowolo, Chukwumah Nneka Maureen, Micheal Abimbola Alade, Maha El Tantawi

**Affiliations:** 1 Department of Child Dental Health, Obafemi Awolowo University, Ile-Ife, Nigeria; 2 Nigeria Institute of Medical Research, Yaba, Lagos State Nigeria; 3 Community Oral Health Department, Tehran University of Medical Sciences, Tehran, Iran; 4 Faculty of Health Sciences, University of Zaragoza, Zaragoza, Spain; 5 Faculty of Dentistry, University of British Columbia, Vancouver, Canada; 6 Obafemi Awolowo University Teaching Hospitals Complex, Ile‑Ife, Nigeria; 7 Department of Preventive Dentistry, School of Dentistry, College of Medical Sciences, University of Benin, Benin, Nigeria; 8 Department of Preventive Dentistry, Alexandria University, Alexandria, Egypt

**Keywords:** Dental caries, Gingivitis, Children, Prevention, Risk factors

## Abstract

**Background:**

There is little is known about the factors associated with caries experience and gingivitis among 6–11-year-old children in Nigeria. The aim of the study was to determine the prevalence and preventive oral health behaviors associated with caries and gingivitis among 6–11-year-old children in Nigeria.

**Methods:**

A cross-sectional questionnaire-based survey was conducted in Ile-Ife, Nigeria. The dependent variables were caries and gingivitis. The dmft/DMFT index was used to assess dental caries experience (present or absent) and caries severity. The gingival index was used to assess the prevalence (present or absent) and severity of gingivitis (healthy gingiva/mild gingivitis versus moderate/severe gingivitis). The independent variables were preventive oral health behaviors (frequency of daily tooth brushing, frequency of consumption of refined carbohydrates in-between-meals, use of fluoridated toothpaste, and use of dental floss, history of dental service utilization). A series of logistic regression analysis models were constructed to determine the associations between the dependent and independent variables after adjusting for confounders (age, sex, and socioeconomic status).

**Results:**

There were 69 (5.2%) children with caries. The mean (standard deviation) dmft was 0.08 (0.457) and the mean DMFT was 0.02 (0.159). There were 839 (63.3%) children with gingivitis with a mean (SD) gingival index score of 0.503 (0.453). Children who seldom or never used fluoride toothpaste had significantly higher odds of developing moderate to severe gingivitis (AOR; 1.671; 95% CI: 1.003–2.786; p = 0.049). Children with middle socio-economic status had significantly lower odds of developing moderate to severe gingivitis (AOR: 0.573; 95%CI: 0.330–0.994; p = 0.048). There were no risk indicators identified for caries.

**Conclusion:**

The prevalence of dental caries was low while the prevalence of gingivitis was high in the study population. The daily used of fluoridated toothpaste seem to reduce the risk for moderate/severe gingivitis. Further studies are needed to understand these findings.

## Introduction

Dental caries and periodontal diseases (especially gingivitis) are the most common dental conditions in children [1]. More than 530 million children globally are affected by caries of the primary teeth; caries affecting the permanent teeth is the most common childhood oral health condition [2]; and gingivitis is nearly a universal finding in children [3]. Caries and gingivitis result from the interaction of various predisposing factors over time; and if untreated, progress to jeopardize oral health, overall health, and the quality of life of affected individuals as their age increases [4]. Yet, both diseases are preventable.

In Nigeria, the prevalence of caries among school-aged children ranges from 13.9 to 17.4% [5–13]. Most of the studies on the prevalence of caries were conducted in urban areas [5–9], with fewer reports from sub-urban [10–12] and rural areas [13]. Data on the risk factors for caries in school-aged children in Nigeria is sparse, and that of gingival health even less. The prevalence of gingivitis ranged from 82.9% in a hospital-based study [14], to 98% in a school based study [15]. School-based studies are not representative of the profile of children in Nigeria because of the high out-of-school population – as high as 40% of children who should be in the primary school and 60% of children who should be in the secondary school are out of school [16]. Hospital based samples are also biased samples as the hospital population do not represent the profile of the general population [17].

To our knowledge, there is no household survey of the oral health of school-aged children in Nigeria. Household surveys are valuable for providing information that is truly representative of the general population especially in low-income countries like Nigeria [18]. Also, preventive oral health interventions for school children in Nigeria have produced mixed results [9] indicating a need for studies to better understand the risk factors associated with poor oral health [19]. The primary objective of this study was therefore, to determine the prevalence of caries and gingivitis among a population of children aged 6–11 years in Nigeria using a household survey. The secondary objective was to determine the associations between preventive oral health behaviors and the prevalence of dental caries and gingivitis. The study’s null hypothesis was that there will be no statistically significant association between preventive oral behaviors and the prevalence of caries and gingivitis in 6–11-year-old children in Nigeria.

## Methods

### Ethical considerations

Ethical approval for the conduct of this study was obtained from the Ethics and Research Committee of the Institute of Public Health, Obafemi Awolowo University, Ile-Ife, Nigeria (IPH/OAU/12/1887). Parental consent was obtained from the parents of children who participated in the study.

### Study design and study population

This cross-sectional survey was conducted in Ife Central Local Government Area of Osun State, a semi-urban community in Southwestern Nigeria. Study participants included children aged 6-11-years whose parents consented to their study participation. Critically ill children and those that could not give independent responses to the survey questions were excluded from study. The data was collected through a household survey conducted from December 2018 to January 2019.

### Sample size and sampling technique

The minimum sample size for the study was calculated with the formula proposed by Araoye [20], using a caries prevalence of 13.9% [10], a 5% margin of error, a study power of 80% and a confidence level of 95%. The minimum sample size was 1233 children.

The children were recruited using a multi-stage sampling technique. For the first stage, 70 of the 700 enumeration areas in Ife Central Local Government Area were selected using the simple random sampling method. For the second stage, every other household in the selected enumeration areas was identified as eligible for participation. For the final stage, one child who met the inclusion criteria was recruited per household for study participation. Households that declined participation were replaced by the next eligible household. In households where there were more than one eligible study participants, the children balloted to identify who would be included in the study. The others were also examined and those who required treatment were referred appropriately, but their data was not collected for the study. Recruitment of participants continued until the minimum study sample size was attained.

### Data Collection

Data were collected using an interviewer administered questionnaire followed by a brief dental examination. The survey instrument was administered by trained field workers with experience in collecting data for national surveys. The field workers and clinicians were trained on the study protocol, the use of the data collection tools, sample selection (including household listing and selection), and the ethical conduct of research [21]. Data collected included confounding, independent and dependent variables.

*Confounding variables*: Data collected from each child included age at last birthday, sex at birth (male, female), and socioeconomic status. Data on socioeconomic status was determined using the mother’s level of education with the father’s occupation [22]. The mother’s level of education was classified as: no formal education, Quranic and primary school education (score 2); secondary school education (score 1) and tertiary education (scored 0). The father’s occupation was also categorized into three levels: civil servants or skilled professionals with a tertiary level of education (score 1); civil servants or skilled professionals with a secondary level of education (score 2); unskilled, unemployed, students, and civil servants or skilled professionals with a primary and or Quranic education (scored 3). The social class of the parents was determined by adding the score of the mother’s level of education to that of the father’s occupation. Each child was allocated into social classes I–V (class I, upper class; class II, upper middle class; class III, middle class; class IV, lower middle class; class V, lower class). When a child had lost a parent, the socioeconomic status was determined using the status of the living parent. For this study, the five classes were re-grouped into three: high (upper and upper-middle classes), middle (middle class) and low (lower- middle and lower classes) socio-economic status. The re-grouping was done in line with a prior index regrouping for children in Nigeria [23].

*Independent variables*: Respondents were asked about their daily frequency of tooth brushing, daily frequency of consumption of refined carbohydrates in-between-meals, daily use of fluoridated toothpaste, daily use of dental floss and the period of the last dental check-up. The acceptable level for each component was set as follows: brushing more than once daily, eating refined carbohydrates in-between-meals less than once daily, use of fluoridated toothpaste always or almost always, flossing at least once a day, and attending a dental check-up within the last year. The study questionnaire was developed by Khami et al. [24] and had been used in previous studies conducted in Nigeria [25, 26].

#### Dependent variables

All participants received a dental examination to determine the caries experience and severity of gingivitis. Each participant was examined by the trained dentists, sitting upright in a chair under natural light, with sterile dental mirrors.

#### Gingivitis

Gingival changes on the mesial, distal, buccal and lingual surfaces of six index permanent teeth (11, 16, 26, 31, 36, and 46) was assessed using the gingival index of Löe and Silness [27] was recorded. When an index tooth was missing, a score was not recorded [28]. The scores for each surface examined ranged from 0 to 3. The sum of the assessment for the four surfaces of each tooth was divided by four to determine the gingival index for each tooth. The gingival indices for the six permanent teeth were added and divided by six to determine the gingival index for each person. All children with scores greater than 0 were classified as having gingivitis. the severity of gingivitis was classified by the gingival index scores into healthy (0), mild (0.1–1), moderate (1.1–2), or severe (2.1–3). Gingivitis was further dichotomized into healthy gingiva/mild gingivitis versus moderate/severe gingivitis for the bivariate and logistic regression analyses [29].

#### Caries experience

The teeth were cleaned of debris and dried using a sterile gauze before assessment for caries. The presence of caries in the primary and permanent teeth was assessed using the decayed (d/D), missing (m/M), and filled (f/F) teeth (t/T) indices respectively [30]. Caries experience was divided into caries experience present (dmft/DMFT > 0) or absent (dmft/DMFT = 0). Caries was recorded as present when there were obvious cavitations on the tooth according to the World Health Organization criteria [31], the tooth was extracted due to caries or there as a tooth restored due to caries. Non-cavitated carious teeth were classified as sound tooth tissue.

### Standardization of examiners

Three qualified dentists undergoing postgraduate residency training in pediatric dentistry, were calibrated on the study protocol and the clinical examination. Training was followed by practice on patients: each clinician examined and scored the child for caries status as prescribed in the study protocol. Results were subjected to a Cohen’s weighted kappa score analysis to determine intra- and inter-examiner variability. The intra- and inter-examiner Cohen’s weighted kappa scores for the three dentists were all greater than 0.80.

### Data analysis

Univariate analyses were calculated as means and standard deviations for numerical variables or frequencies and percentages for categorical variables. Bivariate analysis was conducted using Chi-squared test and ANOVA to determine the independent variables associated with the presence of caries, the mean dmft and DMFT scores, the presence of gingivitis and the mean gingival index scores. A series of logistic regression analysis models were constructed to identify the risk indicators for caries and moderate/severe gingivitis in the study population. Potential risk indicators were introduced in successive logistic regression models: model 1 included the sociodemographic factors, model 2 included the oral health behavior indicators, and model 3 included the oral health behavior indicators while controlling for the sociodemographic factors. The estimated coefficients were expressed as adjusted odds ratios (AOR) and their 95% confidence intervals were calculated. The fit of each model was assessed using Nagelkerke R2 and − 2 log likelihood (− 2LL). IBM SPSS version 28.0 was used for statistical analysis and statistical significance was set at 5%.

## Results

There were 1326 study participants with a mean (standard deviation) age of 8.7 (1.9) years. Of these, 839 (63.3%) children had gingivitis with a mean (SD) gingival index score of 0.503 (0.453); and 69 (5.2%) children had dental caries in the primary and/or permanent teeth with a mean (SD) dmft score of 0.08 (0.457) and a mean DMFT score of 0.02 (0.159).

The majority (62.4%) were of low socio-economic status. As shown in Table 1, 1159 (87.4%), children reported brushing once daily, 1133 (85.4%), always using fluoride toothpaste 1024 (77.2%) irregularly use dental floss daily and 1163 (87.7%) had never visited the dentist. Also 298 (22.5%) of children consumed refined carbohydrates in-between-meals less than once daily.

**Table 1:** Socio-demographic characteristics and prevalence of risk indicators for dental caries and gingivitis among 6–11-year-old children in Nigeria (N = 1326)

	No	Percentage
**Age**		
6 7 8 9 10 11	255 231 248 237 204 151	19.2 17.4 18.7 17.9 15.4 11.4
**Sex**		
Female Male	600 726	45.2 54.8
**Socioeconomic Status**		
Low Middle High	827 325 174	62.4 24.5 13.1
**Frequency of daily tooth brushing**		
Irregular/never Once a day Twice a day or more A few times a week	42 1159 93 32	3.2 87.4 7.0 2.4
**Use of fluoridated toothpaste**		
Always/almost always Often Seldom Not at all/no response	1133 111 39 43	85.4 8.4 2.9 3.3
**Use of floss**		
Irregular/never Once a day Twice a day or more A few times a week No response	1024 140 16 55 91	77.2 10.6 1.2 4.1 6.9
**Frequency of daily consumption of refined carbohydrates in-between-meals**		
Not everyday Once daily Twice daily More than twice daily Rarely/Never/ No response	298 347 346 204 51 80	22.5 26.2 26.1 15.4 3.8 6.0
**Most recent visit to the dental clinic**		
A year ago, or less 1–2 years 2–5 years ago > 5 years ago Never Don’t remember/No response	33 25 13 9 1163 83	2.5 1.9 1.0 0.6 87.7 6.3
**Dental caries experience**		
No caries Caries present	1257 69	94.8 5.2
**Gingivitis**		
Healthy gingiva Mild gingivitis Moderate gingivitis Severe gingivitis	507 713 94 12	38.2 53.8 7.1 0.9
**Total**	**1326**	**100.0**

Table 2 shows that the prevalence of caries was, however, higher among children who reported a dental visit in the preceding 12 months. The prevalence of moderate to severe gingivitis was higher among children with high socio-economic status, those who brush once a day or less, those who seldom or never use fluoridated toothpaste, those who consumed refined carbohydrates in-between-meals once a day or more, -and those who reported a recent dental visit. The child’s caries experience and gingival status were not significantly associated with the any of the independent and confounding variables.

**Table 2:** Associations between the prevalence of dental caries, gingivitis, socio-demographic, oral health related behavior and preventive dental care use among children aged 6–11 years (N = 1326)

	Dental Caries Status			Gingival Status			Total N = 1326 n (%)
Variables	**Absent**	**Present**	**p-value**	**Healthy gingiva/Mild gingivitis**	**Moderate/severe Gingivitis**	**p-value**	
	**N = 1257** **n (%)**	**N = 69** **n (%)**		**N = 1220** **n (%)**	**N = 106** **n (%)**		
**Age**							
6 7 8 9 10 11	238 (93.3) 220 (95.2) 235 (94.8) 221 (93.2) 199 (97.5) 144 (95.4)	17 (6.7) 11 (4.8) 13 (5.2) 16 (6.8) 5 (2.5) 7 (4.6)	0.349	234 (91.8) 215 (93.1) 228 (91.9) 219 (92.4) 187 (91.7) 137 (90.7)	21 (8.2) 16 (6.9) 20 (8.1) 18 (7.6) 17 (8.3) 14 (9.3)	0.977	255 (19.2) 231 (17.40 248 (18.7) 237 (17.9) 204 (15.4) 151 (11.4)
**Sex**							
Female Male	565 (94.2) 692 (95.3)	35 (5.8) 34 (4.7)	0.415	556 (92.7) 664 (91.5)	44 (7.3) 62 (8.5)	0.481	600 (45.2) 726 (54.8)
**Socioeconomic Status**							
Low Middle High	780 (94.3) 313 (96.3) 164 (94.3)	47 (5.7) 12 (3.7) 10 (5.7)	0.369	757 (91.5) 308 (94.8) 155 (89.1)	70 (8.5) 17 (5.2) 19 (10.9)	0.059	827 (62.4) 325 (24.5) 174 (13.1)
**Frequency of daily toothbrushing**							
Once a day or less Twice a day or more	1167 (94.6) 90 (96.8)	66 (5.4) 3 (3.2)	0.517	1131 (91.7) 89 (95.7)	102 (8.3) 4 (4.3)	0.245	1233 (93.0) 93(7.0)
**Use of fluoridated toothpaste**							
Always/Often Seldom/Never	1179 (94.7) 78 (95.3)	65 (5.2) 4 (4.9)	1.00	1148 (92.3) 72 (87.8)	96 (7.7) 10 (12.2)	0.216	1244 (93.8) 82 (6.2)
**Use of dental floss**							
At least once daily Less than once daily	149 (95.5) 1108 (94.7)	7 (4.5) 62 (5.3)	0.813	138 (88.5) 1082 (92.5)	18 (12.5) 88 (7.5)	0.114	156 (11.6) 1170 (88.4)
**Frequency of daily consumption of refined carbohydrates in-between meals**							
One or more times a day Less than once daily	925 (94.7) 332 (95.1)	52 (5.3) 17 (4.9)	0.853	897 (91.8) 323 (92.6)	80 (8.2) 26 (7.4)	0.748	977 (73.7) 349 (26.3)
**Most recent visit to the dental clinic**							
Less than a year ago More than a year	30 (90.9) 1227 (94.9)	3 (9.1) 66 (5.1)	0.534	28 (84.8) 1192 (92.21)	5 (15.2) 101 (7.8)	0.226	33 (2.5) 1293 (97.5)
**Total**	**1257 (94.8)**	**69 (5.2)**		**1220 (92.0)**	**106 (8.0)**		

As shown in Table 3, more children who reported a visit to the dentist within the last year had significantly higher mean DMFT scores (p = 0.043) than those who reported visiting over a year ago. Also, more children who seldom or never used fluoridated toothpaste had significantly higher mean gingival index scores (p = 0.038).

**Table 3:** Associations between the mean DMFT index score, gingival index score, socio-demographic factors, oral health behavior indicators and preventive dental use indicators

Variables	DMFT score				Gingival index score		
	**n**	**Mean**	**SD**	**p-value**	**Mean**	**SD**	**p-value**
**Age**							
6 7 8 9 10 11	255 231 248 237 204 151	0.1294 0.0996 0.0927 0.1519 0.0245 0.0795	0.564 0.554 0.435 0.653 0.155 0.391	0.133	0.486 0.521 0.491 0.504 0.535 0.516	0.562 0.560 0.546 0.542 0.527 0.539	0.935
**Sex**							
Female Male	600 726	0.1250 0.0785	0.589 0.408	0.091	0.479 0.530	0.536 0.554	0.094
**Socioeconomic status**							
Low Middle High	827 325 174	0.1088 0.0708 0.1092	0.526 0.413 0.510	0.488	0.524 0.456 0.524	0.553 0.524 0554	0.162
**Frequency of daily tooth brushing**							
Once a day or less Twice a day or more	1233 93	0.1022 0.0645	0.506 0.385	0.482	0.509 0.491	0.545 0.565	0.766
**Use of fluoridated toothpaste**							
Always/often Seldom/Never	1244 82	0.0997 0.984	0.554 0.488	0.973	0.495 0.584	0.531 0.624	0.038
**Use of dental floss**							
At least once daily Less than once daily	156 1170	0.1154 0.0974	0.601 0.483	0.673	0.584 0.497	0.616 0.536	0.067
**Frequency of daily consumption of refined carbohydrates in-between-meals**							
One or more times a day Less than once daily	977 349	0.1013 0.0946	0.496 0.503	0.827	0.518 0.479	0.553 0.528	0.251
**Most recent visit to the dental clinic**							
Less than a year ago More than a year	33 1293	0.2727 0.0951	1.098 0.473	0.043	0.559 0.506	0.635 0.544	0.593

Table 4 shows that all the models tested for assessing the independent factors associated with caries experience were not statistically significant (p > 0.05). The models explained only a very small proportion of the results obtained (model 1 = 1.0%; model 2 = 0.6%; model 3 = 1.5%;). There was no risk indicator significantly associated with caries experience.

As shown in Table 5, model 3 explained the highest proportion of the occurrence of moderate to severe gingivitis in the study participants (Model 1 = 1.2%; Model 2 = 1.6%; Model 3 = 3.1%). None of the models tested was statistically significant (p > 0.05). Children with middle socio-economic status had significantly lower odds of developing moderate to severe gingivitis compared to children with low socio-economic status (AOR: 0.573; 95%CI: 0.330–0.994; p = 0.048). Children who reported seldom or never using fluoride toothpaste had significantly higher odds of developing moderate to severe gingivitis (AOR; 1.671; 95% CI: 1.003–2.786; p = 0.049).

**Table 4:** Associations between the prevalence of caries, socio-demographic factors, oral health behavior indicators and preventive dental use indicators (N = 1326)

Variables	Model 1 AOR (CI)	p-value	Model 2 AOR (CI)	p-value	Model 3 AOR (CI)	p-value
**Age**	0.913 (0.784–1.063)	0.240			0.916 (0.786–1.068)	0.263
**Sex**						
Female Male	1.000 0.800 (0.492–1.301)	0.369			1.000 0.796 (0.489–1.297)	0.360
**Socioeconomic Status**						
Low Middle High	1.000 1.067 (0.525–2.167) 0.649 (0.339–1.241)	0.386 0.858 0.191			1.000 0.660 (0.344–1.264) 1.067 (0.524–2.171)	0.415 0.210 0.858
**Frequency of daily toothbrushing**						
Twice a day or more Once a day or less			1.000 1.841 (0.560–6.040)	0.315	1.000 1.776 (0.538–5.861)	0.345
**Use of fluoridated toothpaste**						
Always/often Seldom/Never			1.000 0.941 (0.329–2.691)	0.910	1.000 0.917 (0.441–1.903)	0.815
**Use of dental floss**						
At least once daily Less than once daily			1.000 1.247 (0.556–2.799)	0.593	1.000 1.251 (0.556–2.812)	0.588
**Frequency of daily consumption of refined carbohydrates in-between-meals**						
Less than once daily One or more times a day			1.000 1.106 (0.628–1.946)	0.728	1.086 (0.615–1.918) 1.000	0.777
**Most recent visit to the dental clinic**						
Less than a year ago More than a year			1.000 0.467 (0.136–1.610)	0.228	1.000 0.463 (0.133–1.606)	0.225
Nagerkalkes R^2^ -2LL	0.010 537.81	0.350	0.006 539.79	0.783	0.015 535.48	0.802

**Table 5:** Association between the prevalence of moderate/severe gingivitis and socio-demographic factors, oral health behavior indicators and preventive dental use indicators (N = 1326)

Variables	Model 1 AOR (CI)	p-value	Model 2 AOR (CI)	p-value	Model 3 AOR (CI)	p-value
**Age**	1.029 (0.910–1.164)	0.651			1.034 (0.913–1.170)	0.601
**Sex**						
Female Male	1.000 1.177 (0.786–1.762)	0.430			1.000 1.139 (0.758–1.713)	- 0.530
**Socioeconomic Status**						
Low Middle High	1.000 0.601 (0.348–1.039) 1.305 (0.761–2.238)	0.740 0.069 0.334			1.000 0.573 (0.330–0.994) 1.3431 (0.780–2.314)	- 0.048 0.287
**Frequency of daily toothbrushing**						
Twice a day or more Once a day or less			1.000 2.237 (0.794–6.307)	- 0.128	1.000 2.216 (0.753–6.001)	- 0.154
**Use of fluoridated toothpaste**						
Always/often Seldom/Never			1.000 1.572 (0.771–3.206)	- 0.213	1.000 1.671 (1.003–2.786)	- 0.049
**Use of dental floss**						
At least once daily Less than once daily			1.000 0.666 (0.385–1.152)	- 0.146	1.000 0.648 (0.375–1.119)	0.120
**Frequency of daily consumption of refined carbohydrates in-between-meals**						
Less than once daily One or more times a day			1.000 1.194 (0.749–1.903)	- 0.455	1.000 1.216 (0.760–1.945)	- 0.414
**Most recent visit to the dental clinic**						
Less than a year ago More than a year			1.000 0.454 (0.165–1.251)	- 0.127	1.000 0.485 (0.177–1.328)	- 0.159
Nagerkalkes R^2^ -2LL	0.012 729.04	- 0.165	0.016 726.33	- 0.101	0.031 721.35	- 0.155

## Discussion

The study results show that the prevalence of dental caries was low while prevalence of gingivitis was high in this population of 6–11-year-old children in Nigeria. None of the socio-demographic or preventive oral health behaviors explored in the study was significantly associated with the caries experience. The severity of caries was significantly higher for children reporting a dental visit within the last year. Also, none of the socio-demographic or preventive oral health behaviors explored were associated with moderate/severe gingivitis in this study. However, more children who reported always using fluoride toothpaste had significantly higher mean gingival index scores. These results therefore suggest that the null hypothesis for our study can be partially rejected, and the alternate hypothesis partially sustained.

This is the first study in Nigeria to explore the oral health profile of school aged children using a household survey approach. However, the cross-sectional design makes it impossible to infer any cause-effect relationships between the variables. Also, there was the risk for socially desirable responses though the collection of data that required only a single day recall likely reduced the risk for recall bias. Further, family income, a recognized socio-economic risk indicator for dental conditions in children [32] was not assessed in this study. This was because income is a culturally sensitive topic in many Nigerian communities. We however, used a measure of socioeconomic status that had been validated for use in West Africa, and had also been used to determine the socioeconomic status in for multiple oral health studies in Nigeria. Despite these limitations, the study findings can be used to develop appropriate community-based preventive interventions for reducing the two most globally prevalent dental conditions in school-aged children in countries with similar socioeconomic profile like Nigeria.

Like previous studies on caries prevalence [5–8, 13, 20] and gingivitis [14, 15] in school age children in Nigeria, our results confirm that the prevalence of caries is low and the prevalence of gingivitis is high among school children. The prevalence of both diseases, however, were lower than prior reports. The lower prevalence reported in the current study may be attributable to the use of a household survey unlike prior reports that used school-based or hospital-based surveys. This finding may indicate that school-based or hospital-based studies could overestimate the prevalence of dental conditions in children. The low population-level caries prevalence when compared to the prevalence of school-based studies caries may indicate that distinct school-based and community-based caries control plans are both needed to further reduce the caries prevalence in this population [33].

Community-based approaches for caries prevention produce better results [34] and may therefore be more appropriate for this community. This is because of the evidence indicating a symptomatic approach to dental care seeking by the children in this population: a previous dental visit had significantly higher mean caries experience score. Promoting anti-caries school policies [35, 36] and facilitating prompt diagnosis and treatment of caries lesions through school and community health programs [33, 37] may be valuable strategic approaches for moving towards a caries elimination agenda for school-aged children in the study community and other similar populations.

We further found that children who seldom/never used fluoridated toothpastes had higher odds of moderate/severe gingivitis. Their mean gingival index score was also significantly higher. An earlier study had indicated that fluoride containing toothpastes reduce the risk of gingival inflammation [38] indicating the possible dual direct protective role of fluoridated toothpaste (fluoridated toothpaste is primarily used to prevent dental caries) [39]. These findings suggest that the high proportion of children who always/often use of fluoridated toothpaste may explain the low prevalence of caries among the study participants, as the decline in caries had been associated with the use of topical fluoride especially in dentifrices [39]. Yet, despite the high use of fluoridated toothpaste, the prevalence of gingivitis was very high but the severity was low. The high prevalence of gingivitis despite the use of fluoridated toothpaste may be the result of the very high proportion of children who brush once a day. Twice daily tooth brushing eliminates the risk for gingivitis [40]. On the contrary, despite the inadequacy of the frequency of daily tooth brushing, the daily tooth brushing practice may explain the low prevalence of severe gingivitis. There however, may be a role that fluoride plays in reducing the risk for gingivitis as daily toothbrush frequency in itself was not significantly associated with a lower prevalence of gingivitis. Further studies are needed to explore our postulations from these study findings. Studies are also needed to understand how fluoride containing toothpastes affect gingival health.

Finally, we found no association between other preventive oral health behaviors and dental caries or gingivitis. The non-significant association between the frequency of consumption of refined carbohydrate in-between-meals and the prevalence of caries differs from previous evidence on this association in early childhood [41]. The threshold used to determine caries risk for the study population was established in a prior study, which identified that children who consumed refined carbohydrates once a day or more were at risk of developing caries in early childhood [42]. This threshold was defined however, for hospital patients and may be a less sensitive threshold for population-level survey conducted among school-aged children.

The non-significant association between the use of dental floss and the prevalence of gingivitis or the gingival index score is unusual as dental floss is an effective tool for reducing the risk of gingivitis. We found no association in this study population. This is the first study determining the prevalence of use of dental floss and the association of its use with gingivitis prevalence in a school-age population in Nigeria. An earlier study had determined the association of its use with the prevalence of caries in children in Nigeria and found no significant association [43], and another study found no significant association between its use and the prevalence of caries and gingivitis in adolescents in Nigeria [44]. The non-statistically significant association between the use of dental floss and gingivitis in children in Nigeria needs to be investigated further.

The non-significant association between the dental service utilization and the prevalence of caries is also not unusual. Prior studies had indicated that dental service utilization is often for curative purposes in the study population just like it is for hospital utilization for care [45–47]. Utilization of dental services for preventive care reduces the risk for caries and gingivitis because it facilitates the early detection and prompt management of these diseases [48]. The high cost of out-of-pocket expenses for health care services [47, 49] may be a barrier to the routine use of health care service in Nigeria. Oral health care is very expensive [19], yet over 60% of the population live below poverty line [50] and access to health insurance is poor [51]. It is important to study policy implications of this finding, in order to identify how to improve oral health care service utilization to reduce the risk for poor oral health especially gingivitis, and reduce the oral and general health risks associated with poor oral health [52]. This will likely be important in countries with low to middle incomes, such as Nigeria.

## Conclusion

The prevalence of dental caries was low while the prevalence of gingivitis was high in the study population. No common risk factor was identified for caries and gingivitis though the poor use of fluoride containing toothpaste appears to increase the risk for gingivitis in the study population. While the study did not find an association between the use of fluoride containing toothpaste and caries experience (possibly due to the low caries experience), it is important to explore the possible role of the regular use of fluoride containing toothpaste as a common risk approach to reduce the risk for gingivitis and further reduce the prevalence of caries in the study population.

## Data Availability

All data generated for this study are presented in the manuscript. The dataset for the study can however be accessible on reasonable request from one the study author, Morenike Oluwatoyin Folayan, toyinukpong@yahoo.co.uk.

## Abbreviations

AOR: Adjusted Odds Ratio.
CI: Confidence Interval.
SD: Standard Deviation.
dmft/DMFT: Decay, missing, filled teeth.
